# Replacing discontinued Big Tech mobility reports: a penetration-based analysis

**DOI:** 10.1038/s41598-023-28137-7

**Published:** 2023-01-17

**Authors:** Francesco Finazzi

**Affiliations:** Department of Economics, University of Bergamo, Bergamo, Italy

**Keywords:** Socioeconomic scenarios, Scientific data

## Abstract

People mobility data sets played a role during the COVID-19 pandemic in assessing the impact of lockdown measures and correlating mobility with pandemic trends. Two global data sets were Apple’s Mobility Trends Reports and Google’s Community Mobility Reports. The former is no longer available online, while the latter is no longer updated since October 2022. Thus, new products are required. To establish a lower bound on data set penetration guaranteeing high adherence between new products and the Big Tech products, an independent mobility data set based on 3.8 million smartphone trajectories is analysed to compare its information content with that of the Google data set. This lower bound is determined to be around 10^−4^ (1 trajectory every 10,000 people) suggesting that relatively small data sets are suitable for replacing Big Tech reports.

## Introduction

People mobility data received considerable attention during the COVID-19 pandemic when they were used to assess the impact of lockdown measures^1–7^ and to understand the correlation between mobility patterns and pandemic trends^8–14^. In a smart society, the importance of mobility data is not limited to the pandemic. In general, mobility is affected by global long-term events such as economic crises^15–17^ and conflicts^18^, and by local short-term events like social unrest and extreme natural events^19–22^. Mobility data are also used to better assess exposure to health-threatening phenomena^23^.

Smartphone mobility data play a key role in estimating mobility patterns^24–30^. Apple’s Mobility Trends Reports and Google’s Community Mobility Reports were two global data sets made available to researchers during COVID-19 pandemic^31–35^. The first data set was discontinued on April 14, 2022^36^, while the second is still available but no longer updated after October 15, 2022^37^.

Here, a global mobility data set derived from the Earthquake Network (EQN) citizen science initiative^38^ is analysed to assess the feasibility of making available to the scientific community mobility products which, in their information content, are similar to Apple and Google products. Since 2012, EQN implements the first smartphone-based earthquake early warning system^39,40^ allowing citizens to receive real-time alerts directly on their smartphones when seismic waves are incoming. To join the initiative, people install the EQN smartphone app which turns the smartphone into a seismic detector. Once installed, the app regularly collects the smartphone location which is needed by the earthquake detection algorithm^41^ and to first alert people who are close to the epicentre at the time of the earthquake. As of today, more than 8 million people joined the initiative globally making EQN one of the most active citizen science projects.

The data set provided by EQN includes 6.1 billion location data points collected by its smartphone app from March 11, 2020, to September 22, 2022. From the data set, 3.8 million anonymized spatio-temporal smartphone trajectories are reconstructed and used to produce mobility metrics at the country level. Contrary to Big Tech data sets, which benefits from a high penetration among the population, the analysed data set is relatively small. Thus, the methodology adopted to analyse the data set plays a key role in estimating metrics characterized by the lowest possible bias and accompanied by a measure of uncertainty. Also, the methodology addresses peculiar aspects of smartphone-based location data, such as: the non-negligeable uncertainty on smartphone coordinates, missing data and the non-homogeneous geographical penetration of smartphone apps in the population.

Similarly to Gao et al^3^, two metrics are provided: daily average travel distance (M_1_) and the percentage of people who did not move during the 24 h of the day (M_2_). Analysis is restricted to a group of 17 countries for which uncertainty on the estimated mobility metrics is small enough to allow reasonable comparisons between countries and/or different periods: Argentina (ARG), Chile (CHL), Colombia (COL), Costa Rica (CRI), Ecuador (ECU), Greece (GRC), Guatemala (GTM), Italy (ITA), Mexico (MEX), Nicaragua (NIC), Panama (PAN), Peru (PER), Philippines (PHL), Slovenia (SVN), Turkey (TUR), the United States (USA) and Venezuela (VEN).

Metrics M_1_ and M_2_ are provided at daily temporal resolution and at different levels of temporal smoothing, uncertainty included. Mobility metrics time series are correlated with time series of Google’s product in order to compare their mutual information content and to assess a lower bound on the number of smartphone trajectories (with respect to the country population size) which guarantees a high adherence between products.

Mobility data analysed in this work are elaborated daily by EQN and regularly made available on Zenodo through the MobMeter^42^ data set. To the best of our knowledge, MobMeter is currently the only open source mobility data set covering multiple global countries and an extensive time period up to present. MobMeter is thus a unique instrument for researchers who may benefit from including mobility patterns as covariates in their statistical or stochastic models, with applications in multiple fields including epidemiology, climatology and economics.

## Results

### Global long-term mobility trends

**Figure 1:**
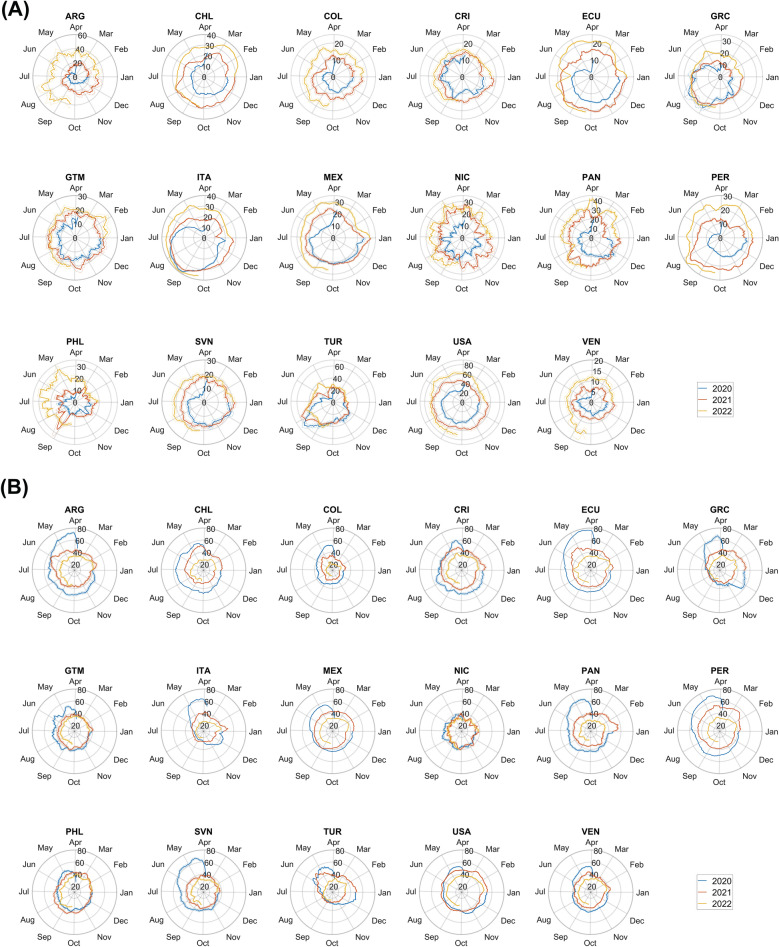
(**A**) Estimated smoothed mobility metric M1 (daily average travelled distance) and (**B**) estimated smoothed mobility metric M2 (percentage of people who did not move during the 24 h of the day) based on a 14-day moving average from March 24, 2020, to September 22, 2022. M1 is expressed in kilometres while M2 in percentage. Dashed lines are 95% confidence bands.

Mobility metrics and their smoothed versions are computed for the aforementioned countries. Figure 1 shows polar plots of M_1_ and M_2_ time series based on a 14-day temporal smoothing. All countries show significant decrements in the M_1_ metric and significant increments in the M_2_ metric during the few months after March 11, 2020 (initial phase of the COVID-19 pandemic). Differences between countries are observed in the temporal rapidity of the subsequent “recovery”. Some exhibit “lobed” polar plots. This is the case for GRC, ITA and TUR, which show fast recoveries during the summer of 2020 and a contraction of people mobility during the subsequent winter. All other countries exhibit “spiralling” polar plots, which is a sign of a slow recovery. This behaviour is clear for South American countries like ARG, COL, PER and VEN.

### Short-local events in mobility metrics

**Figure 2:**
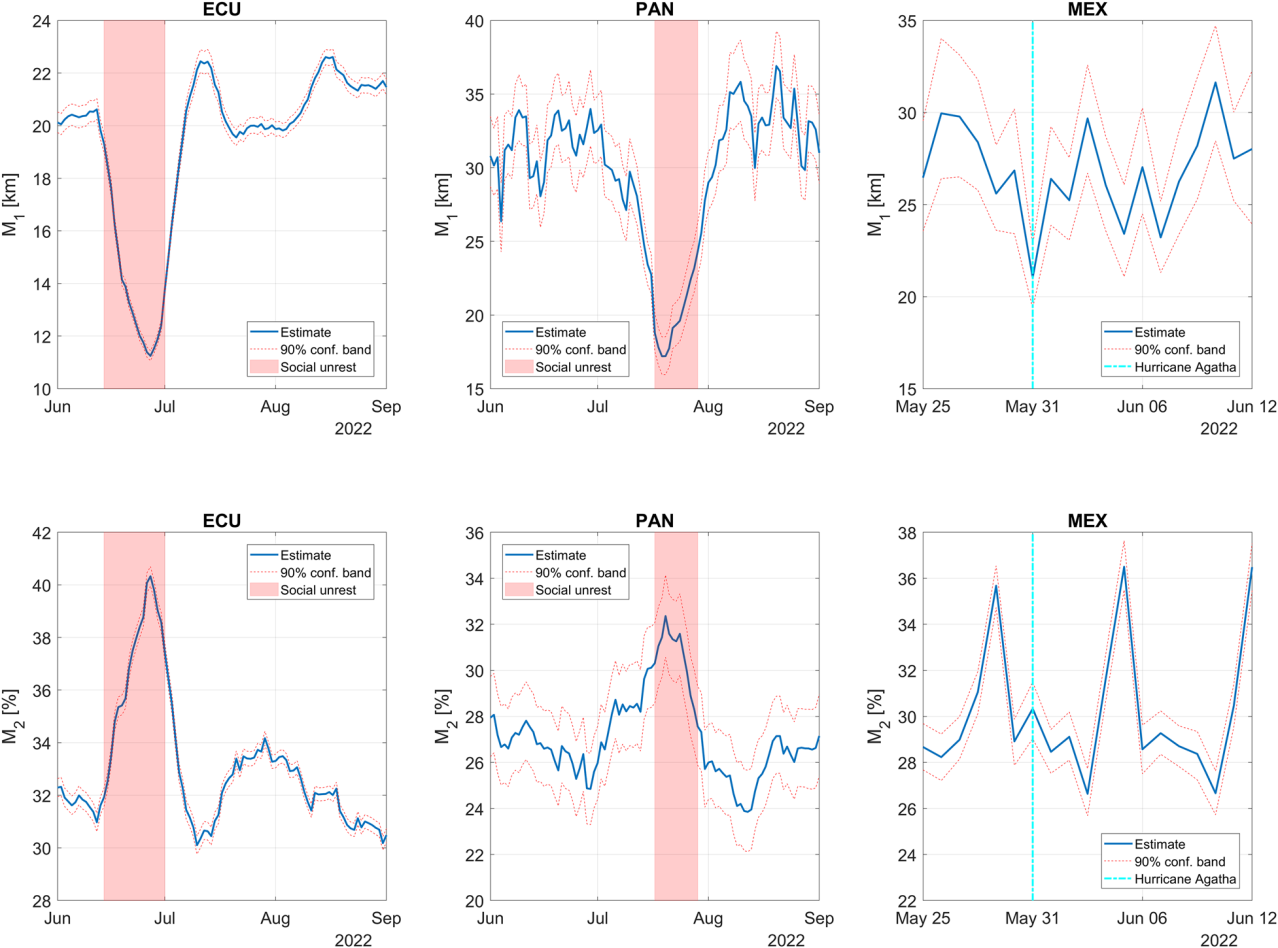
Variations in mobility metrics M_1_ and M_2_ during 2022 social unrest in ECU and PAN and when Hurricane Agatha struck Oaxaca, MEX. For ECU and PAN, mobility metrics are based on 7-day smoothing. For MEX, no smoothing is applied.

M_1_ and M_2_ time series are also affected by short-term local events. Figure 2 shows how mobility metrics significantly changed during social unrest in ECU and PAN and when a hurricane made landfall in MEX. In ECU, social protests^43^ occurred between June 13 and June 30. M_1_ dropped from approximately 20 km to 11.5 km, while M_2_ raised from approximately 32% to 40%. In PAN, social protests^44^ broke out on July 16 and lasted nearly two weeks. M_1_ dropped from around 30 km to 17 km, while M_2_ raised from approximately 27% to 32%. Between May 30 and 31, 2022, Hurricane Agatha hit the Oaxaca state of MEX with flooding and mudslides that killed at least 10 people and left 20 missing^45^. M_1_ dropped from an average of around 28 km (on the previous days) to around 22 km. This drop is seen at the country level, so it was mitigated by the relatively small area (with respect to the area of MEX) impacted by the hurricane. M_2_ did not change significantly with respect to the previous day.

### Comparison with Google’s Community Mobility Reports

A comparison is made between metrics M_1_ and M_2_ and the mobility indices of the Google product. Google’s mobility indices are given as variations (in percentage) in the number of visits to categories of places with respect to a baseline. This means that Google’s indices do not carry exactly the same information of M_1_ and M_2_, nor the unit of measure is the same. Nonetheless, all indices are based on smartphone spatio-temporal trajectories, and their linear correlation is expected to be medium–high.

M_1_ is compared with Google’s “Transit stations”, “Parks” and “Retail and recreation” indices while M_2_ with Google’s “Residential” and “Workplaces” indices. It is expected that “Transit stations”, “Parks” and “Retail and recreation” indices positively correlate with the daily average distance travelled by people, and that the “Residential” and the “Workplaces” indices correlate (positively and negatively, respectively) with the percentage of people who did not move during the 24 h of the day.

**Figure 3:**
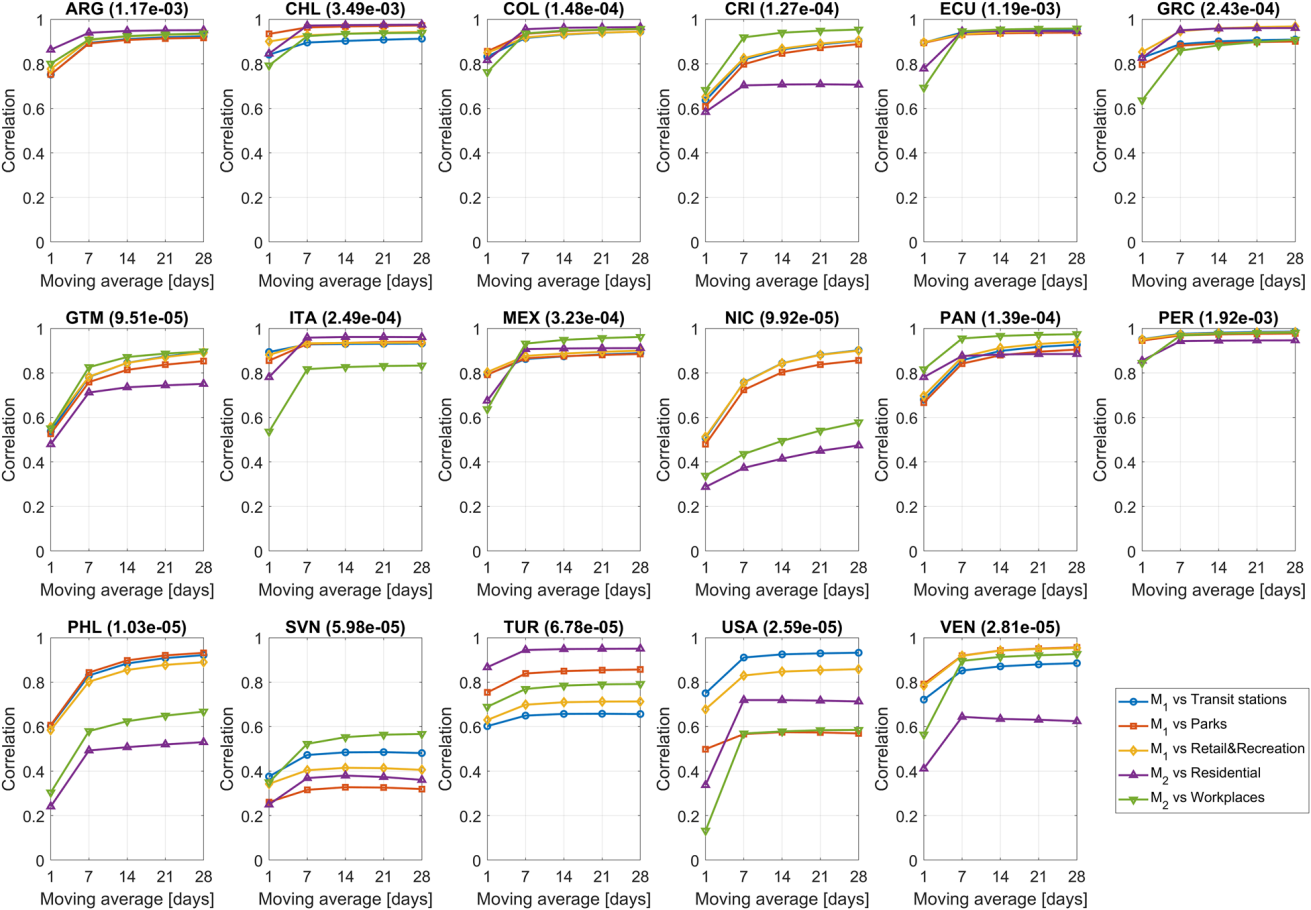
Linear correlation between the M_1_ mobility metric and Google’s “Transit stations” index, between M_1_ and Google’s “Parks” index, between M_1_ and Google’s “Retail and Recreation” index, between the M_2_ mobility metric and Google’s “Residential” index and between the M_2_ and Google’s “Workplaces” index. 1-day moving average means no smoothing. All correlations between M_2_ and Google’s “Workplaces” index are negative but they are reported without sign to assure readability of the plots. In brackets, the average data set penetration in each country.

**Figure 4:**
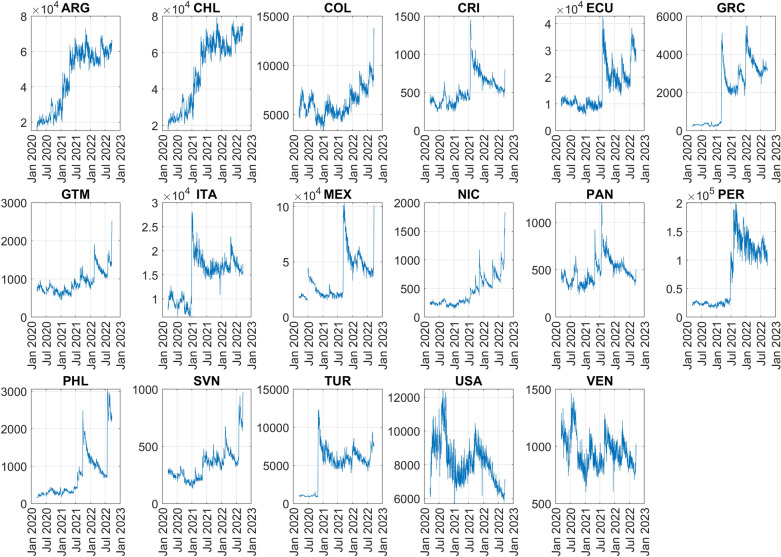
Time series of the number of daily smartphone trajectories used to estimate M_1_ and M_2_ metrics by country.

Figure 3 shows, for each country and for different levels of smoothing of the time series, the linear correlation between the metrics and Google’s indices. For most countries, correlations are high and significantly increase when moving from no smoothing to a 7-day moving average smoothing. The lowest correlations are exhibited by NIC, SVL and PHL, which are among the countries with the lowest average number of daily smartphone trajectories (average sample size) in the data set (see Fig. 4). Robustness of above correlations is tested in the Methods section.

### Data set penetration analysis

The relationship between correlations and average sample size is better described by accounting for country population size. The average sample size is computed for each country for the period from March 11, 2020, to September 22, 2022, and divided by country population size. This gives the average data set penetration in each country.

**Figure 5:**
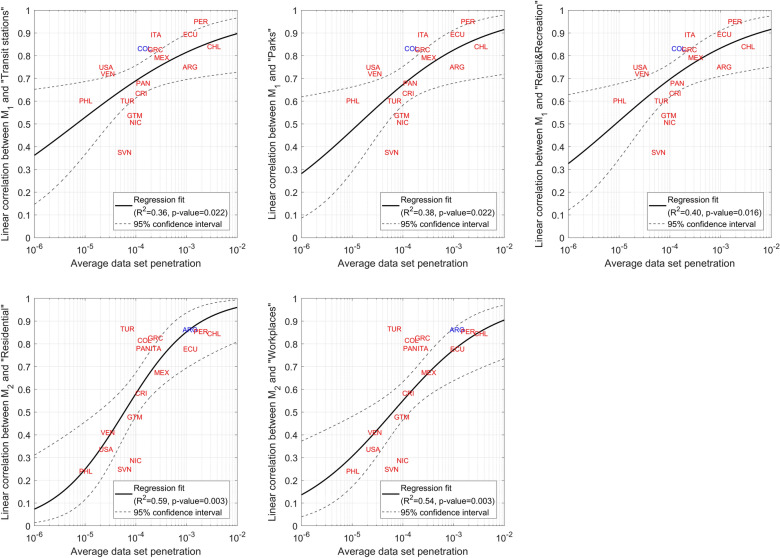
Linear correlation between M_1_ and M_2_ and Google’s mobility indices vs average data set penetration for the 17 countries under the non-smoothing case. Solid lines are the fits by a beta regression model with logit link function, while dashed lines are 95% confidence intervals on fitted values. R^2^ are pseudo coefficients of determination, while the p-values refer to an F-test on the regression model, which tests whether the model fits significantly better than a model with only the constant term. Each label is a country. Overlapping labels are in different colours. All correlations between M_2_ and Google’s “Workplaces” index are negative but they are modelled without sign to keep consistency with Fig. 3 and to allow beta regression.

Under the non-smoothing case, for each correlation between metrics and indices the 17 average penetration values are related with the 17 correlations using a beta regression (see Methods section). Figure 5 shows the data and modelling results. In general, the higher the average penetration, the higher the correlation between indices. Additionally, average penetrations between around 10^−4^ (i.e., 1 smartphone trajectory every 10,000 people) delimit the transition between medium (~ 0.5) to high (> 0.7) correlations.

## Discussion

Mobility metrics and indices are proxies of societal health and stress. Their constant monitoring informs assessment of the impact of both long- and short-term adverse events. Thus, location data collected by smartphone apps play an important role, as they carry information on people mobility. With Apple’s Mobility Trends Reports discontinued and Google’s Community Mobility Reports no longer updated, it is crucial to understand whether similar products can be produced from alternative mobility data sets and made available to the scientific community.

This study reconstructs smartphone trajectories from an independent mobility data set provided by the Earthquake Network citizen science initiative. These allow estimation of country-level mobility metrics with relatively high correlations to similar indices by Google. The average data set penetration among the country population requires 1 smartphone trajectory for every 10,000 people. This prove that relatively small data sets are suitable for replacing country-level Big Tech products when their analysis is based on a sound methodology which also provides measures of uncertainty.

Since it happened with Google’s and Apple’s products, it is meaningful to discuss the possible end-of-life of the MobMeter data set. While there is no guarantee that the Earthquake Network initiative will last years or decades, it is likely that citizens' interest in earthquakes and in the EQN smartphone app will not vanish in the foreseeable future. On the other hand, personal mobility data are inevitably connected to the privacy and the security of the individual^46^. The collection of personal data by smartphone apps is usually regulated by the app-store owner, with Google and Apple, again, as the main actors. This implies that new rules and limits on how personal data are collected may come into place at any time, making the publishing of MobMeter hard or impossible (long before Earth’s seismicity will fade away).

## Methods

### Trajectory description

The mobility data set analysed in this work includes smartphones trajectories collected from the 17 countries during the period from March 11, 2020, to September 22, 2022 (926 days). Each trajectory covers any possible subset of the 926 days (from only one day to the full period).

In general, the $k$ th trajectory, $1\le k\le K$, is composed of ${M}_{k}$ observations, with $K$ the total number of trajectories. The $m$th observation, $1\le m\le {M}_{k}$, of the $k$th trajectory is given by.

1 $$\left({ID}_{k}, {lat}_{m}, {lon}_{m}, {u}_{m}, {t}_{m}\right),$$

where ${ID}_{k}$ is the anonymized smartphone/trajectory identifier, ${lat}_{m}$ and ${lon}_{m}$ are the latitude and longitude smartphone coordinates, ${u}_{m}$ is the uncertainty on smartphone coordinates, and ${t}_{m}$ is the timestamp.

Uncertainty ${u}_{m}$ is in metres and represents the standard deviation (sigma) of two independent normal distributions centred on each smartphone coordinate. Timestamp ${t}_{m}$ is based on the country local time and refers to the time ${lat}_{m}$ and ${lon}_{m}$ are observed by the smartphone. Timestamps have the following constraint: $min\left({t}_{m+1}-{t}_{m}\right)\ge 20$ min.

### Trajectory sanitation

Trajectory observations which exhibit a latitude and longitude equal to zero are removed from the trajectory. Zero values are returned by a smartphone when geolocation is not possible. Also, ${u}_{m}$ values equal to zero or negative are replaced with an uncertainty equal to 25 m. This is the typical uncertainty when a smartphone localizes itself using Wi-Fi networks and/or cell phone antennas. The percentage of trajectory observations affected by the replacement is 0.08%.

### Daily estimates of M_1_ and M_2_ metrics

M_1_ and M_2_ mobility metrics are estimated with daily temporal resolution. For each day $d=1,\dots ,926$, only trajectories with at least 12 observations between 00:00:00 and 23:59:59 local time and with a temporal span of at least 12 h between the first and the last observation contribute to the estimate of M_1_ and M_2_. At the $d$th day and the $i$th country, the number of daily trajectories that satisfy the constraints above is denoted by ${N}_{d,i}$. In general, ${N}_{d,i}\ne {N}_{{d}^{{\prime}},i}$ for $d\ne {d}^{{\prime}}$.

For any two consecutive trajectory observations, the geodetic distance ${l}_{m,m+1}$ between $\left({lat}_{m},{lon}_{m}\right)$ and $\left({lat}_{m+1},{lon}_{m+1}\right)$ is computed. The following transformation is then applied:

2 $${\widehat{l}}_{m,m+1}=\left\{\begin{array}{cc}{l}_{m,m+1}& if\,\, {l}_{m,m+1}\ge {u}_{m}+{u}_{m+1} \\ 0& otherwise\end{array}.\right.$$

The transformation implies that the estimated travelled distance ${\widehat{l}}_{m,m+1}$ between time ${t}_{m}$ and ${t}_{m+1}$ is greater than zero only if the 1-sigma uncertainty disks do not overlap. The daily travelled distance by the $k$th smartphone is given by:

3 $${\widehat{L}}_{d,i,k}=\sum_{m=1}^{{M}_{k}}{\widehat{l}}_{m,m+1}I\left({t}_{m}\in \left[\left.{\tau }_{d},{\tau }_{d+1}\right)\right.\right),$$

where $I\left({t}_{m}\in \left[\left.{\tau }_{d},{\tau }_{d+1}\right)\right.\right)$ is 1 if timestamp ${t}_{m}$ is within day $d$ and 0 otherwise (with ${\tau }_{d}$ the timestamp related with midnight of day $d$). Since ${\widehat{l}}_{{M}_{k},{M}_{k}+1}$ is computed using the last trajectory observation of day $d$ and the first of day $d+1$, Eq. (3) implies that any travel occurring across midnight contributes to the total travelled distance on day $d$.

Moreover, let

4 $${\widehat{U}}_{d,i,k}=\left\{\begin{array}{cc}1& if\,\, {\widehat{L}}_{d,i,k}<0.2 \,\, km \\ 0& otherwise\end{array}\right.$$

be a binary variable equal to 1 if the smartphone does not move during the 24 h of the day and 0 otherwise. The threshold 0.2 $\mathrm{km}$ is set to accommodate for indoor smartphone movements during the day that may sum up to a small distance.

For each country, first-level administrative divisions (regions) are considered. Each region ${R}_{i,c}$ is described in terms of multiple polygons defined in the geographical space. These polygons are freely available for download at diva-gis.org/gdata. The number of first-level administrative divisions by country is given in Table 1.

**Table 1:** Number of first-level administrative divisions by country.

Country	Number of first-level administrative divisions
Argentina	24
Chile	16
Colombia	32
Costa Rica	7
Ecuador	24
Greece	8
Guatemala	22
Italy	20
Mexico	32
Nicaragua	18
Panama	13
Peru	26
Philippines	81
Slovenia	14
Turkey	81
United States	52
Venezuela	25

The daily average travelled distance for the $c$th region of the $i$th country is given by:

5 $${\widehat{L}}_{d,i,c}=\frac{1}{{N}_{d,i,c}}\sum_{k=1}^{{N}_{d,i}}{\widehat{L}}_{d,i,k} \cdot I \left(\left({\overline{lat} }_{k}, {\overline{lon} }_{k}\right)\in {R}_{i,c}\right),$$

where $I\left(\left({\overline{lat} }_{k},{\overline{lon} }_{k}\right)\in {R}_{i,c}\right)$ is equal to 1 if the daily average smartphone coordinates $\left({\overline{lat} }_{k},{\overline{lon} }_{k}\right)$ are in the ${R}_{i,c}$ region, and 0 otherwise. ${N}_{d,i,c}$ is the number of daily trajectories in the $c$th region.

Let ${p}_{i,c}$ be the population count of the $c$th region, $1\le c\le {C}_{i}.$ The daily average distance for the $i$th country (mobility metric M_1_) is given by

6 $${\widehat{L}}_{d,i}=\sum_{c=1}^{{C}_{i}}{\widehat{L}}_{d,i,c}\cdot {w}_{i,c},$$

where

7 $${w}_{i,c}=\frac{{p}_{i,c}}{{\sum }_{c=1}^{{C}_{i}}{p}_{i,c}},$$

is a weight based on the region population. The adoption of this weighting approach is dictated by three reasons: (1) the spatial distribution of smartphone-app users does not necessarily mimic the population distribution; (2) events affecting people mobility may be limited to some regions, or their strength vary across regions^47^; (3) in general, a weighting approach based on a population stratification helps reduce the bias of estimates^48^.

By replacing ${\widehat{L}}_{d,i,k}$ with ${\widehat{U}}_{d,i,k}$ in Eq. (5) and following the same procedure described above, the mobility metric M_2_ (i.e., ${\widehat{U}}_{d,i}$) is computed for each day and country.

### Uncertainty assessment

Uncertainty on daily M_1_ and M_2_ figures (i.e., ${\widehat{L}}_{d,i}$ and ${\widehat{U}}_{d,i}$, respectively) is assessed using a non-parametric bootstrap approach^49^. At the $b$th bootstrap iteration, $1\le b\le B$, values ${\widehat{L}}_{d,i,h,b}$ and ${\widehat{U}}_{d,i,h,b}$, ${1\le h\le N}_{d,i,c}$, are sampled with replacements from the observed ${\widehat{L}}_{d,i,k}$ and ${\widehat{U}}_{d,i,k}$ values restricted to the $c$th region. Following Eqs. (5)–(7), the resampled values are used to produce the bootstrap sample $\left({\widehat{L}}_{d,i,1},\dots ,{\widehat{L}}_{d,i,B}\right)$ and the bootstrap sample $\left({\widehat{U}}_{d,i,1},\dots ,{\widehat{U}}_{d,i,B}\right)$. Fixing $B=1000$, bootstrap samples are used to compute their empirical distribution. This allows evaluation of $\left(100-\alpha \right)\%$ bootstrap confidence intervals^50^ on the ${\widehat{L}}_{d,i}$ and ${\widehat{U}}_{d,i}$ estimates, with $\alpha$ equal to 5 in this work.

### Temporal smoothing

Temporal smoothing of $\left\{{\widehat{L}}_{d,i}\right\}$ and $\left\{{\widehat{U}}_{d,i}\right\}$ time series is based on a $q$-day moving average, with $q$ equal to 7, 14, 21 and 28. The smoothed version of ${\widehat{L}}_{d,i,c}$ is

8 $${\widehat{L}}_{d,i,c}^{q}=\frac{1}{\sum_{s=d-q+1}^{d}{N}_{s,i,c}}\sum_{s=d-q+1}^{d}\sum_{k=1}^{{N}_{s,i}}{\widehat{L}}_{s,i,k}\cdot I\left(\left({\overline{lat} }_{k},{\overline{lon} }_{k}\right)\in {R}_{c}\right).$$

Similarly, ${\widehat{U}}_{d,i,c}^{q}$ is defined by replacing ${\widehat{L}}_{s,i,k}$ with ${\widehat{U}}_{s,i,k}$ in Eq. (8). Confidence intervals on ${\widehat{L}}_{d,i,c}^{q}$ and ${\widehat{U}}_{d,i,c}^{q}$ are based on bootstrap samples which include $q$ days of resampled data. This allows obtaining confidence intervals with the correct width.

### Comparison with Google’s Community Mobility Reports

Community Mobility Reports by Google gives percentages of variation in the number of visits to place categories with respect to a baseline. The categories are “Retail and recreation”, “Grocery and pharmacy”, “Parks”, “Transit stations”, “Workplaces” and “Residential”. For each category, time series of percentages of variation are available at both country and regional levels with daily temporal resolution.

M_1_ and M_2_ time series are compared with Google’s country-level time series by computing linear correlations. M_1_ is correlated with “Transit stations”, “Parks” and “Retail and recreation” indices while M_2_ with “Workplaces” and “Residential” indices. Comparison is made using both non-smoothed and smoothed time series (i.e., $q\in \left\{7, 14, 21, 28\right\}$). A highly positive or highly negative correlation means that M_1_ and/or M_2_ carry information on people mobility similar to that of Google’s community mobility reports.

### Robustness analysis

**Figure 6:**
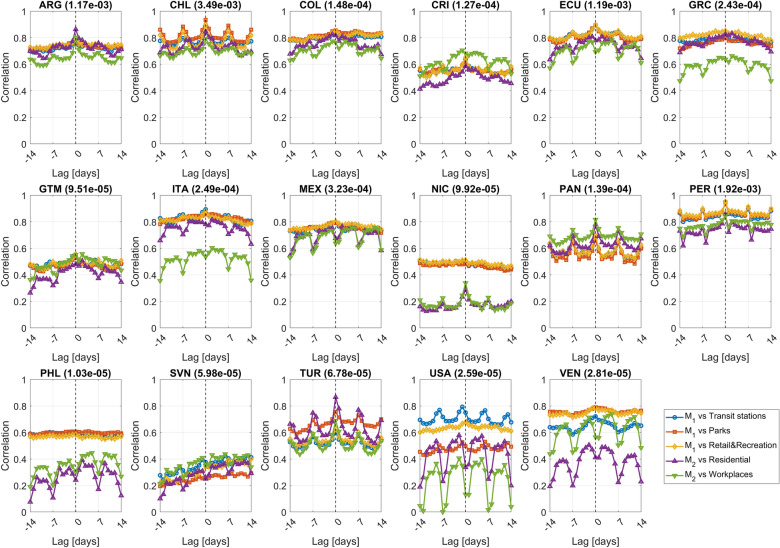
Time-shifted correlation graphs for correlations computed between the M_1_ and M_2_ metrics and the Google’s indices.

To test robustness of the comparison described in the previous section, a time-shifted correlation analysis is also implemented. Whenever two time series are considered, one time series is shifted by a lag of $\Delta =-14,\dots ,0,\dots ,14$ days and linear correlation is computed. Figure 6 shows results for the 17 countries.

For countries with a relatively high average data set penetration (see for instance ARG, CHL and PER), correlations are maximum when $\Delta =0$ and, due to the weekly cycle, they also tend to be high when $\Delta =-14,-7, 7, 14$. When penetration is lower this behaviour is disrupted and the maxima are not necessarily at $\Delta =0$ (or not all of them are at $\Delta =0$). TUR is an exception since peaks of the time-shifted correlation graph are located where expected despite the low penetration.

### Beta regression on correlations vs average penetration

A beta regression is adopted to describe the relationship between the average data set penetration and the correlations without sign $\left|\rho \right|$ between the non-smoothed M_1_ and M_2_ metrics and Google’s indices. Beta regression is imposed by $\left|\rho \right|\in \left[\mathrm{0,1}\right]$.

For the generic $i$th country, $\left|{\rho }_{i}\right|\sim \mathcal{B}\left({\mu }_{i},\phi \right)$, where $\phi$ is the precision parameter of the beta distribution and $g\left({\mu }_{i}\right)={{\varvec{x}}}_{i}{^{\prime}}{\varvec{\beta}}$, with $g$ the logit link function, ${{\varvec{x}}}_{i}$ the vector of regressors and ${\varvec{\beta}}$ the vector of unknown model parameters. Here, ${{\varvec{x}}}_{i}=\left[1,{log}_{10}\left({\pi }_{i}\right)\right]{^{\prime}}$, with ${\pi }_{i}$ the average data set penetration for the $i$th country. Model fitting capability is described by the pseudo coefficient of determination ${R}^{2}={corr\left(\left|\rho \right|,\widehat{\left|\rho \right|}\right)}^{2}$, with $\widehat{\left|\rho \right|}$ the model estimate. An F-test on the regression model is used to tests whether the model fits significantly better than a model with only the constant term (i.e., ${{\varvec{x}}}_{i}$ = 1).

### Sensitivity analysis

Estimates of M_1_ and M_2_ are based on three arbitrary choices. First, only trajectories with at least $n$ observations within a span of at least $n$ hours are used ($n=12$). Second, ${\widehat{l}}_{m,m+1}={l}_{m,m+1}$ only if ${l}_{m,m+1}\ge {ru}_{m}+{ru}_{m+1}$ ($r=1$, see Eq. (2)). Third, ${\widehat{U}}_{d,i,k}=1$ if ${\widehat{L}}_{d,i,k}<z$ ($z=0.2 \mathrm{km}$, see Eq. (4)).

The choice of $n$ affects both M_1_ and M_2_, while the choice of $r$ and $z$ only affects M_2_. Values used in this work are the result of a sensitivity analysis. Considering ITA and the period from March 11, 2020, to September 22, 2022, the correlation without sign $\left|{\rho }_{1}\right|$ between M_1_ (non-smoothed) and the “Transit stations” index by Google and the correlation without sign $\left|{\rho }_{2}\right|$ between M_2_ (non-smoothed) and the “Residential” index by Google are estimated for each combination of $n\in \left\{3, 6, 9, 12, 15\right\}$, $r\in \left\{1, 2, 3\right\}$ and $z\in \left\{0.1, 0.2, 0.3, 0.4\right\} \mathrm{km}$.

Considering all combinations, $\left|{\rho }_{1}\right|$ ranges between 0.878 and 0.893, while $\left|{\rho }_{2}\right|$ ranges between 0.766 and 0.781. Correlations are not significantly affected by large variations in $n$, $r$ and $z$. For both $\left|{\rho }_{1}\right|$ and $\left|{\rho }_{2}\right|$, the maximum is reached when $n=12$, $r=1$ and $z=0.2 \mathrm{km}$.

## Data Availability

The MobMeter data set which includes the mobility metrics produced in this work is available on Zenodo at https://zenodo.org/record/6984638. Google’s Community Mobility Reports are available at https://www.google.com/covid19/mobility/.
